# Real‐world prognostic factors for survival among treated patients with metastatic pancreatic ductal adenocarcinoma

**DOI:** 10.1002/cam4.4415

**Published:** 2021-11-22

**Authors:** Kenneth H. Yu, Muhammet Ozer, Paul Cockrum, Andy Surinach, Shu Wang, Bong Chul Chu

**Affiliations:** ^1^ Memorial Sloan Kettering Cancer Center and Weill Cornell Medical College New York New York USA; ^2^ Capital Health Medical Center Trenton New Jersey USA; ^3^ Ipsen Cambridge Massachusetts USA; ^4^ Genesis Research Hoboken New Jersey USA

**Keywords:** antineoplastic agents, electronic health records, pancreatic ductal adenocarcinoma, prognostic factors, real‐world evidence, treatment options

## Abstract

**Background:**

Many real‐world studies of patients with metastatic pancreatic ductal adenocarcinoma (mPDAC) are restricted to single centers, limiting the generalizability of their insights. This study aimed to identify important population‐based predictors for survival in patients diagnosed with mPDAC in a broader setting.

**Methods:**

Data between 1 January 2017 and 31 December 2019 were extracted from the Flatiron Health EHR database. Treatment‐specific predictive models were generated for patients treated with first‐line gemcitabine+nab­paclitaxel (GNP), FOLFIRINOX, gemcitabine monotherapy (gem‐mono), and second‐line liposomal irinotecan‐based regimens. The holdout method was used for cross‐validation. Age at diagnosis, sex, BMI, smoking status, and ECOG performance score were included in all models with additional demographic, clinical characteristics, and hematological function assessed for inclusion.

**Results:**

Of the 3625 patients, 43% received GNP, 26% received FOLFIRINOX, 7% received gem‐mono, and 23% received other regimens; 40% (*n* = 1448) advanced to the second line. Among all first‐line patients, the following were included in the final model: prior surgery, white blood cell (WBC) counts, serum albumin (SA), liver function tests (LFTs), serum bilirubin, serum carbohydrate antigen 19–9, and ascites. Models for patients receiving specific therapies differed from the overall model, GNP (ascites removed), FOLFIRINOX (stage at initial diagnosis added), and gem‐mono (LFTs omitted). Alkaline phosphatase (ALP), SA, and WBC counts were important predictors of survival among patients treated with second‐line liposomal irinotecan. Across all regimens, the strongest predictors of survival were ECOG score, SA, and ALP.

**Conclusions:**

In this real‐world study of patients with mPDAC, important population prognostic factors of survival were identified in a large cohort of patients receiving systemic treatment.

## Graphical abstract

This retrospective observational analysis of US patient‐level electronic health record data identified a sample of 3625 patients who initiated first‐line systemic therapy for metastatic pancreatic ductal adenocarcinoma from 2017 to 2019. Important population prognostic factors of survival were identified that applied across all regimens including performance status, serum albumin, and alkaline phosphatase levels. Additional patient characteristics were identified as prognostic factors of survival in the context of specific therapies.

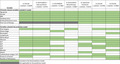

**Lay summary**

This study used Flatiron Health, a large research database of health records from patients with cancer across the United States. The records to not contain personal identifiers to ensure patient anonymity. The study analyzed all patients who began treatment for metastatic pancreatic cancer in between 1 January 2017 and 31 December 2019 and used information on medical history, test results, and cancer treatments received. This is the largest study of its kind to date and the findings that ECOG performance score, liver function, and serum albumin predict survival may help to inform clinical practicec.

## 1 INTRODUCTION

Pancreatic cancer ranks in 11th place for cancer incidence in the United States in 2020, comprising about 3% of cases.^1^, ^2^ However, it is the third leading cause of cancer mortality. Specific early symptoms are generally lacking^3^, ^4^ contributing to delays in diagnosis with fewer than 20% of patients having resectable disease at diagnosis. The disease has aggressive biology characterized by early dissemination and intrinsic tumor resistance to radiation and chemotherapy. These together account for the poor prognosis of pancreatic cancer, which has an estimated 5‐year survival rate of 10% for all diagnoses and as low as 3% for patients initially diagnosed with metastatic disease.^2^, ^4^ Pancreatic ductal adenocarcinoma (PDAC) is the most common type of pancreatic cancer, comprising approximately 80% of new cases.^5^ It is characterized by extensive stromal tissue that promotes a microenvironment for cancer progression^6^ and resistance to chemotherapy and radiation treatment,^7^ while also forming a barrier to drug delivery.^8^

Gemcitabine monotherapy (gem‐mono) was shown in the late 1990s to result in longer survival compared with existing regimens. Subsequent to this, a combination of gemcitabine with nanoparticle albumin‐bound paclitaxel (GNP) was found to produce further improvements in survival in metastatic PDAC (mPDAC).^9^ A combination regimen consisting of 5‐FU/leucovorin plus oxaliplatin and irinotecan (FOLFIRINOX) has also been shown to improve survival compared with gem‐mono.^10^, ^11^ For second‐line and later use in mPDAC, liposomal irinotecan in combination with 5‐FU/leucovorin demonstrated improved overall survival (OS) when compared with 5‐FU/leucovorin alone in the phase 3, NAPOLI‐1 randomized trial.^12^, ^13^ These findings led to FDA approval for patients with mPDAC with documented progression after gemcitabine or gemcitabine‐based therapy.^12^ Furthermore, liposomal irinotecan is the only therapy so far to have National Comprehensive Cancer Network (NCCN) Category 1^14^ and American Society of Clinical Oncology (ASCO)^15^ recommendations as a post‐gemcitabine therapy. No specific recommendations currently exist for third‐line therapy.^16^

There are only limited data on real‐world treatment outcomes in mPDAC, and data from clinical trials, which conform to strict eligibility criteria, are not representative of real‐world practice. Real‐world studies reported in the literature have included estimates of the prognostic impact of patient and disease characteristics in patients receiving systemic therapy for mPDAC.^17^, ^18^, ^19^, ^20^, ^21^ Such studies have been limited in sample size due to a reliance on one or a limited number of study centers or a focus on patients receiving a particular drug regimen. This has limited their usefulness for guiding decision‐making in the treatment of patients with mPDAC. Here, we report a large retrospective analysis of electronic health records of patients with mPDAC in the United States and the development of a validated predictive model for survival based on routinely collected data (demographic, clinical, and laboratory parameters), with the aim of improving the understanding of patient care in mPDAC in community oncology settings.

## 2 METHODS

### 2.1 Data source and study design

This retrospective observational cohort analysis used data from the Flatiron Health database, a longitudinal, demographically diverse database derived from de‐identified electronic health record data. The Flatiron database includes data from over 280 cancer clinics or approximately 800 sites of care, and the distribution of patients between community and academic practices largely reflects patterns of care in the United States.^22^, ^23^ Patient‐level data include structured data such as laboratory values, treatments, and diagnosis codes. Documents providing unstructured data in Flatiron are curated via technology‐enabled abstraction; these include clinician notes, radiology reports, and death notices. Informed consent was waived as the study was retrospective and used only routinely collected data. To ensure patient privacy and confidentiality Flatiron de‐identifies all data it collects and delivers, and this includes provisions to prevent re‐identification.

### 2.2 Study population

Patients included in the data source were those with a diagnosis code for pancreatic cancer (International Classification of Diseases, Ninth Revision, Clinical Modification (ICD‐9‐CM): 157.x or ICD‐10‐CM: C25.x), two documented clinical visits, on separate days, on or after 1 January 2014, had a pathology consistent with adenocarcinoma of the pancreas, and were diagnosed with stage IV disease or were diagnosed with earlier stage pancreatic cancer and subsequently developed recurrent or progressive disease on or after 1 January 2014. Patients were included based on a diagnosis of mPDAC between 1 January 2017 and 31 December 2019 as included in the January 2021 delivery of Flatiron data. To be eligible, patients also had pathology consistent with mPDAC. Other requirements were ≥18 years of age and a recorded activity such as a visit or treatment within 90 days on or after the diagnosis. Patients were also required to have received a treatment and to have a recorded activity after the start date of therapy. Patients were excluded if the date of death (uniformly assigned as the 15th of the month of death for OS) preceded the treatment start date.

### 2.3 Lines of therapy

Lines of therapy in the Flatiron database are defined operationally and not necessarily the same as clinically defined lines of therapy. The index date for each patient was defined as the first day of the initial systemic therapy that began after diagnosis of mPDAC; any prior adjuvant or neoadjuvant therapies were not included in the definition. All additional components of therapy in the first 28 days were considered part of the same line of therapy. A treatment line was considered to have advanced to the next if a new drug was added after 28 days with the following exceptions, which could be made within 90 days of the start of therapy: 5‐FU substituted for capecitabine or vice versa; leucovorin substituted for levoleucovorin or vice versa; LV/levoleucovorin added; or protein‐bound paclitaxel added to a gemcitabine regimen or vice versa. Chemoradiation therapy was not included in the analysis.

### 2.4 Variable definitions

Baseline variables were evaluated for this analysis, including demographics (age, height, weight, body mass index, smoking status, index year, sex, race, and region), clinical characteristics (stage, site of primary tumor, ECOG performance status (PS) score, prior surgery, ascites, sites, and number of metastases), and laboratory values (neutrophils, lymphocytes, WBC, serum albumin [SA], alanine transaminase [ALT], aspartate transaminase [AST], alkaline phosphatase [AP], lactate dehydrogenase [LDH], serum bilirubin [SA], neutrophil–lymphocyte ratio, and serum carbohydrate antigen 19–9 [CA 19–9]). The presence of ascites and metastatic sites was identified based on diagnosis records in the EHR with relevant ICD‐9‐CM/ICD‐10‐CM codes. Lab values were categorized into normal, abnormal, and unknown based on their reported values and normal ranges. Clinical and laboratory characteristics were included if taken within +/‐60 days of the index date and the reading closest to index date used for patients with multiple readings (the most severe was used for readings taken on the same day). The primary endpoint was OS, defined as the time between the index date and date of death. Censoring was applied at the last activity date if no death occurred. Univariate Cox proportional hazard models were used to estimate the association of OS with age category, sex, BMI category, prior or no/not known surgery, cancer stage (IV, I–III, and other), ECOG PS score, presence of ascites, number of metastases (0/not captured/1/≥2), and lab and hematologic values (normal/abnormal/unknown). All estimates were conducted for each line sequential line of therapy (first, second, and third lines) and for the regimens GNP, FOLFIRINOX, gem‐mono, and (for second and third lines only) liposomal irinotecan/5‐FU leucovorin. Categorical variables with missing data were included in the models.

### 2.5 Statistical methods

All analyses were performed separately for first‐, second‐, and third‐line treatments. Descriptive statistics were employed for continuous variables, including number of patients, mean, median, standard deviation, interquartile range, minimum, and maximum. Comparisons of continuous variables were made using the *t*‐test of mean or nonparametric Wilcoxon rank‐sum test of median as appropriate. Categorical variables were described as percentages and the Chi‐squared tests or the Fisher exact tests were used for comparisons. The Kaplan–Meier methods were used to derive time to death and median time for OS with 95% confidence intervals (CIs). Univariate Cox proportional hazards models were used to derive hazard ratios (HRs) and 95% CIs for death and time to treatment failure based on the listed variables, by determining whether an individual variable was associated with an outcome and assessing its magnitude and statistical significance. Key variables known to be important prognostic factors as well as any others found to be significant at a level of 0.2 were then carried forward to a multivariate Cox regression model. In the final models, variables were selected as covariates using a stepwise variable selection procedure to develop a good predictive model of OS. The model included important variables (HR <0.9 or >1.1) as well as any others according to a statistical significance threshold of <0.15 for inclusion and <0.1 for subsequent stepwise selection.

The log hazards (regression coefficients) or HRs with 95% CIs were determined. Proportional hazards testing of each of the risk factors in the multivariable model and log cumulative hazard curves by log of time were used to determine whether effects were constant over time.

A holdout model was used for cross‐validation purposes, and data were divided into training (70%) and validation/test (30%) datasets. Predictive accuracy of the final OS model was assessed on the validation/test dataset based on Harrel's and Uno's concordance statistics and time‐dependent receiver operating characteristic (ROC) curves for dealing with the right‐censored data. Using the final model, the distribution of risk factors in the top 10% and bottom 10% of patients ranked by adjusted OS probabilities was also investigated with the aim of characterizing patients at the highest and lowest risk of death. All data analyses were performed using SAS 9.4 (Cary, NC) /R 4.0.0, and a *p* value of <0.05 was specified as the threshold for statistical significance.

## 3 RESULTS

There were 3625 patients included in the study population. All received first‐line therapy on or after diagnosis of metastatic disease. Of these, 1448 (40%) advanced to second‐line therapy, including 230 (16%) who received liposomal irinotecan‐based regimens. Of the second‐line‐treated patients, 504 (34.8%) received third‐line therapy. Demographic and clinical characteristics of patients who began first‐line therapy and patients who began second‐line therapy with liposomal irinotecan/5‐FU leucovorin are shown in Table 1. At diagnosis, 66% of patients in the overall sample had stage IV disease and 64% had ECOG PS scores of 0 or 1. Similar numbers of patients in enrollment years 2017, 2018, and 2019 entered first‐line treatment; however, later‐enrolled patients, especially those from 2019, were more likely to advance to a subsequent line of therapy. The most common racial categories in the overall sample were White (66%), Other (14%), and Black (8%), and similar proportions were found across regimens and treatment lines. All patient groups, whether by treatment line or therapy, included slightly more men than women.

**TABLE 1:** Baseline patient demographic characteristics

Characteristic	1L Gemcitabine+nab­paclitaxel, *N* = 1569	1L FOLFIRINOX, *N* = 959	1L Gemcitabine monotherapy, *N* = 266	2L Liposomal irinotecan, *N* = 230
Age at metastatic diagnosis, Mean (SD), median (IQR)	69 (9), 70 (63, 76)	64 (9), 64 (58, 70)	74 (9), 76 (68, 81)	69 (9), 70 (64, 76)
Age categories at metastatic diagnosis, *n* (%)				
<60 years old	245 (16%)	308 (32%)	22 (8.3%)	31 (13%)
60–69 years old	508 (32%)	390 (41%)	55 (21%)	81 (35%)
70–79 years old	607 (39%)	240 (25%)	98 (37%)	90 (39%)
>=80 years old	209 (13%)	21 (2.2%)	91 (34%)	28 (12%)
Body height (cm), Mean (SD), median (IQR)	169 (11), 168 (160, 178)	171 (11), 173 (163, 178)	168 (10), 168 (160, 175)	169 (10), 170 (163, 177)
Body weight (kg), Mean (SD), median (IQR)	74 (18), 72 (61, 85)	78 (19), 77 (64, 88)	71 (18), 69 (57, 80)	72 (16), 72 (61, 80)
BMI, *n* (%)				
Normal weight	645 (41%)	367 (38%)	126 (47%)	106 (46%)
Underweight	102 (6.5%)	38 (4.0%)	16 (6.0%)	14 (6.1%)
Overweight	470 (30%)	329 (34%)	73 (27%)	69 (30%)
Obese	327 (21%)	217 (23%)	40 (15%)	37 (16%)
Missing	25 (1.6%)	8 (0.8%)	11 (4.1%)	4 (1.7%)
Treatment initiation year, *n* (%)				
2017	488 (31%)	257 (27%)	84 (32%)	25 (11%)
2018	539 (34%)	289 (30%)	91 (34%)	78 (34%)
2019	495 (32%)	387 (40%)	81 (30%)	83 (36%)
2020	47 (3.0%)	26 (2.7%)	10 (3.8%)	44 (19%)
Gender, *n* (%)				
Male	827 (53%)	575 (60%)	144 (54%)	132 (57%)
Female	742 (47%)	384 (40%)	122 (46%)	98 (43%)
Race, *n* (%)				
White	1011 (64%)	628 (65%)	165 (62%)	153 (67%)
Black or African American	153 (9.8%)	67 (7.0%)	28 (11%)	21 (9.1%)
Asian	32 (2.0%)	15 (1.6%)	2 (0.8%)	6 (2.6%)
Other race	197 (12%)	163 (17%)	34 (13%)	31 (13%)
Unknown	176 (11%)	86 (9.0%)	37 (14%)	19 (8.3%)
Region, *n* (%)				
Northeast	243 (15%)	134 (14%)	40 (15%)	38 (17%)
Midwest	184 (12%)	115 (12%)	34 (13%)	31 (13%)
South	722 (46%)	421 (44%)	121 (45%)	101 (44%)
West	235 (15%)	129 (13%)	41 (15%)	40 (17%)
Unknown	185 (12%)	160 (17%)	30 (11%)	20 (8.7%)

### 3.1 Treatment outcomes

Table 2 summarizes the regimens received. GNP was the most widely used first‐line treatment, and FOLFIRINOX the second most widely used. The “other regimens” categories were the most common second‐ and third‐line therapies, which included combinations regimens FOLFIRI and FOLFOX, and other systemic agents. Liposomal irinotecan was a first‐line therapy in a few patients (2.3%) but was a more widely used second‐ or third‐line therapy (16% and 23%, respectively). Median OS (95% CI) during first‐line therapy was 6.5 months (6.1–7.0) with GNP, 9.5 months (8.6–10.3) with FOLFIRINOX, and 3.9 months (3.2–5.1) with gem‐mono. Hazard ratios obtained from univariate and multivariate survival models are shown in Table S1. Clinical and laboratory variables showing prognostic significance for OS in most treatment categories included BMI, disease stage, prior surgery, ECOG PS score, presence of ascites, abnormal serum CA 19–9, and abnormal values for hematology variables and liver function markers. In the multivariate Cox regression model, which controlled for confounding between the variables retaining statistical significance and effect sizes were BMI (with underweight associated with a greater HR), ECOG PS score (2+ vs. 0), WBC, and SA (abnormal values for either associated with a greater HR). Baseline disease state did not have a prognostic effect in second lines of therapy. Prior regimen did not influence prognosis in patients receiving a second or third line of therapy (data not shown).

**TABLE 2:** Most common metastatic treatment regimens by line of therapy

Characteristic	First line *N* = 3625	Second line *N *= 1448	Third line *N* = 504
Regimen, *n* (%)			
Gemcitabine+nab­paclitaxel	1569 (43%)	477 (33%)	56 (11%)
FOLFIRINOX	959 (26%)	153 (11%)	36 (7.1%)
Gemcitabine monotherapy	266 (7.3%)	46 (3.2%)	12 (2.4%)
Liposomal irinotecan	84 (2.3%)	230 (16%)	118 (23%)
Other regimens	747 (21%)	542 (37%)	282 (56%)

### 3.2 Predictive accuracy of final models

**FIGURE 1:**
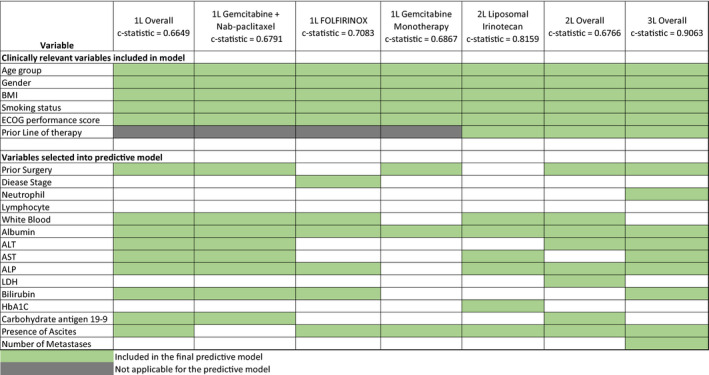
Prognostic models were obtained from multivariable Cox regression model. Models were selected based on univariable *p* value = 0.15 to allow a variable in the model and *p* value = 0.1 to keep a variable in the model. Exception: The 2L GNP cohort model was based on a *p* value =0.1 to allow a variable in the model and *p* value = 0.1 to keep the variable in the model. For models including ALT and AST simultaneously, final model included only ALT. 1L, 2L, and 3L first, second, and third lines of therapy; ALP alkaline phosphatase; ALT, alanine transaminase; AST, aspartate transaminase; BMI, body mass index; ECOG, Eastern Cooperative Oncology Group; Hb1AC, glycosylated hemoglobin; LDH, lactate dehydrogenase; mPDAC, metastatic pancreatic ductal adenocarcinoma

The predictors of the final model for patients receiving first, second, and third lines of therapy are summarized in Figure 1. For patients receiving the four most common first‐line therapies, the final model included the five variables selected for clinical significance plus prior surgery, WBC counts, SA, LFTs (ALP and ALT), serum bilirubin, CA 19–9, and ascites (c‐statistic = 0.66). The model for patients treated with GNP differed from the overall model in that ascites was removed (c‐statistic = 0.68). Stage at initial diagnosis was included in the model for patients treated with FOLFIRINOX, AST/ALT, and CA19‐9, and prior surgery was removed (c‐statistic = 0.68). Among patients treated with gem‐mono, none of the three liver function LFTs, bilirubin, and CA1 9–9 were included in the model (c‐statistic = 0.69). ALP, SA, AST, presence of ascites, HbA1C, and WBC counts were the variables retained in the model in patients treated with second‐line liposomal irinotecan‐based regimens (c­statistic = 0.81).

The bottom and top deciles of patients ranked by predicted OS probability based on characteristics at index date are shown in Table S2. Younger age, female sex, overweight BMI, and ECOG PS scores or 0–1, were all more prevalent in the top decile, whereas some older age categories, patients of underweight BMI, patients with baseline ECOG PS scores of 2, and patients with ascites comprised greater percentages of the bottom decile. Baseline history of surgery not involving the head of the pancreas or history of Whipple surgery was more frequent in the top decile. No consistent patterns were seen for any hematologic variables. Laboratory test results normal for SA, alkaline phosphatase, and glycosylated hemoglobin were more prevalent in the top decile compared with a greater prevalence of abnormal values for these in the bottom decile. First‐line African American patients and patients of other races receiving first‐line therapy were equally represented in the highest and lowest deciles of OS probability. However, for individual treatments, these racial groups comprised a relatively high percentage of the favorable prognosis patients in the GNP group compared to White patients, while in the groups receiving FOLFIRINOX and gem‐mono, the opposite was the case.

Differences in the highest and lowest OS probability deciles in patients entering second‐line therapy showed comparable differences to those for first‐line therapy for the variables of BMI, sex, smoking history, disease stage, prior therapy, and ECOG PS score, presence of ascites, alkaline phosphatase, and SA. African American patients and patients of other races were overrepresented in the highest decile of OS probability compared to White patients.

## 4 DISCUSSION

We performed a retrospective analysis of a large US nationally representative cohort of patients treated for mPDAC and identified prognostic factors for OS based on data from routinely collected electronic medical records. We believe this is the largest study of its kind covering the recent time span (2017–2019) in which all currently FDA‐approved chemotherapeutic regimens, including second‐line liposomal irinotecan‐based regimens, have been in place. Because of the large size of the cohort, it was possible to overcome limitations imposed by subgroup size and to identify prognostic factors in patients receiving specific treatment regimens. In the current study, we found that patient characteristics of known clinical significance (age, sex, BMI, smoking status, and ECOG PS score) were independent prognostic factors in the large overall sample with 3625 patients who received first‐line therapy. To our knowledge, this is the first study of its kind to investigate prognostic effects for OS in patients with mPDAC across multiple subgroups by treatment regimen received as given in real‐world settings. Real‐world median OS with first‐line GNP, FOLFIRINOX, and second‐line liposomal irinotecan‐based therapy was, 6.5, 9.5, and 5.3 months, respectively, which was slightly less than the OS reported in phase 3 randomized controlled clinical trials of 8.5, 11.5, and 6.2 months, respectively, with these regimens.^9^, ^10^, ^12^ The somewhat longer OS reported in clinical trials is not unexpected given the predefined inclusion and exclusion criteria that apply. We also found that baseline ECOG PS score, SA, and ALP were the strongest predictors of OS across almost all regimens.

Previously published real‐world studies have identified some of the variables included in our comprehensive models. Serum C‐reactive protein (CRP), performance status, and CA 19–9 have been identified by a number of small, retrospective real‐world studies as strong predictors of OS.^24^, ^25^, ^28^

Age and PS were identified as independent prognostic variables in a chart review of 154 patients.^26^ A small study of 94 patients in China receiving chemotherapy from 2009 to 2017^29^ identified lymph node involvement, LDH, CA 19–9, CRP, and SA as independent prognostic factors for OS. The type of treatment is often not accounted for in these studies. One study used prospectively collected data from five Japanese hospitals from 2001 to 2013 from patients receiving gemcitabine‐based chemotherapy for nonresectable pancreatic cancer.^27^ Based on univariate and multivariate analyses, they derived a predictive nomogram for survival probability that included age, sex, tumor size, regional lymph node metastasis, and distant metastasis. Song et al. conducted a large population‐based study using the Surveillance, Epidemiology, and End Results (SEER) database to analyze 53,028 patients diagnosed with PDAC from 2004 to 2014.^30^ They used significant prognostic factors for constructing a nomogram based on Cox regression analyses. Their nomogram identified the eight variables of age, race, tumor location, marital status, tumor size, TNM stage, tumor grade, and surgery for predicting cancer‐specific survival. Taking a different approach, Rochefort et al. conducted a matched‐pair analysis of 47 long‐term (≥18 months) survivors of mPDAC with 47 control patients from the same center (Centre Leon Bérard, Lyon, France) between January 2010 and June 2015.^31^ Multivariate analysis found that neutrophil–lymphocyte ratio was the only remaining prognostic factor for long‐term OS in a logistic multivariate model that used backward selection. For prognostic estimates from clinical trials, an analysis of NAPOLI‐1 showed that for patients treated with liposomal irinotecan, mostly as second line of therapy, PS, SA, time since most recent anticancer therapy, tumor stage at diagnosis, liver metastases, and baseline CA19‐9 were prognostic for OS.^13^ A systematic review also identified age, PS, and CA19‐9 as the main prognostic factors across different clinical trials of systemic therapy regimens.^32^

Underweight by BMI, a history of smoking, and ECOG PS score >0 were associated with an adverse risk for OS. Female sex, age <70 years, and prior tumor resection were associated with favorable risk. In contrast, obesity at mPDAC diagnosis appeared to be favorable for OS in patients undergoing second‐line therapy (HR 0.75, 95% CI 0.63–0.89). This is consistent with other reports of obesity as protective in advanced cancer and may be due to a greater nutritional reserve or to the exclusion of patients with baseline signs of cachexia. BMI may also be a surrogate measure of a loss of muscle mass suggestive of cachexia that would indicate a poor prognosis if present at baseline. Disease stage (I, II, and III vs. IV) did not appear as an independent prognostic factor in most treatment categories. SA (normal vs. low) was independently associated with OS for all treatment groups, and this is consistent with many other observational studies of cancer.^33^ SA can be a marker of overall nutritional status, liver function, or a marker of a systemic response to malignant disease. As a prognostic factor, SA has the advantage of being inexpensive widely used in clinical practice. Elevated ALP was also associated with poor prognosis and was the most effective of the markers of metabolism assessed in this study. This enzyme plays a part in bone and liver metabolism.

We constructed prognostic models based on risk factors identified in the Flatiron cohort, which are all readily obtainable in the course of clinical practice. The c‐statistics for the overall population who received first‐, second‐, and third‐line treatments were 0.6649, 0.6766, and 0.7681, respectively, and were greater for the individual treatment categories. This would imply a greater prognostic accuracy than the c‐statistic of 0.6 estimated for American Joint Committee on Cancer (AJCC) staging system (eighth edition).^34^ This suggests that real‐world data from electronic health records might be further developed as a way for physicians to be better informed for the prognosis of an individual patient at the time of diagnosis and be able to initiate more individualized management of mPDAC.

There are several limitations in a study of this type. The structured data are frequently in the form of diagnosis codes and may not capture all comorbid conditions. Real‐world evidence also has a greater frequency of incorrect or missing data than would be the case in a clinical trial. Data collection frequency is not standardized, unlike in a clinical trial, which can lead to unavoidable statistical biases. Labs and performance scores may not be captured in the data due to lack of clinical importance (i.e., normal ECOG PS appear as missing) or site‐specific practices and thus those with missing data may have their outcomes influenced by factors not directly related to their clinical characteristics. Data regarding ascites and sites of metastases were underreported and their role in patient outcomes may not be fully captured in our models. Care received outside of the oncology practice may not be reported back to the EHR and thus acute care episodes (e.g., hospitalizations, emergency room visits) are not accounted for in our models. Missing data were included in the models to account for these potential patterns of care. OS was slightly less than clinical trials of the same treatment, nevertheless, this is likely to be an expected estimate because some patients who would have been ineligible for clinical trials do get treated in clinical practice. The need to prevent re‐identification can also obscure relevant data. For example, all patients ages >85 in the Flatiron databases are included as 85 years of age. Finally, we used only an internal sample from Flatiron for validation and did not compare using external data.

## 5 CONCLUSIONS

In this large real‐world study of patients with mPDAC we have identified prognostic factors of OS in patients receiving contemporary, systemic treatments. There was evidence of variability in these predictors depending on the line of therapy, and the class of systemic therapy received. Prognostic variables identified may help to inform treatment selection and expectations for clinicians. Additional validation studies may be useful in understanding the generalizability of our results.

## CONFLICT OF INTEREST

KHY receives research funding from Ipsen and Bristol Myers Squibb; MO reports no conflict of interest; PC is an employee and has stock in Ipsen; AS and SW are employees of Genesis Research which receives research funding from Ipsen; BCC was an employee of Genesis Research at the time of the study.

## ETHICS STATEMENT

The data accessed were de‐identified in accordance with the HIPAA Privacy Rule, and no personal health information was extracted. Therefore, the study did not require informed consent or institutional review board approval.

## PATIENT CONSENT

Informed consent was waived as this was a non‐interventional study using routinely collected data. The data are de‐identified and subject to obligations to prevent re‐identification and protect patient confidentiality.

## Supporting information

Table S1

Table S2

## Data Availability

The data that support the findings of this study have been originated by Flatiron Health, Inc. These de‐identified data may be made available upon request, and are subject to a license agreement with Flatiron Health; interested researchers should contact <DataAccess@flatiron.com>to determine licensing terms.
